# Transcriptomic analysis and molecular docking reveal genes involved in the response of *Aedes aegypti* larvae to an essential oil extracted from Eucalyptus

**DOI:** 10.1371/journal.pntd.0009587

**Published:** 2021-07-16

**Authors:** Ivana Sierra, Jose Manuel Latorre-Estivalis, Lucila Traverso, Paula V. Gonzalez, Ariel Aptekmann, Alejandro Daniel Nadra, Héctor Masuh, Sheila Ons

**Affiliations:** 1 Laboratorio de Neurobiología de Insectos. Centro Regional de Estudios Genómicos. Facultad de Ciencias Exactas, Universidad Nacional de La Plata, La Plata, Argentina; 2 Centro de Investigaciones de Plagas e Insecticidas (CIPEIN-UNIDEF/CITEDEF/CONICET), Buenos Aires, Argentina; 3 Department of Marine and Coastal Sciences, Rutgers University, School of Environmental and Biological Sciences, New Brunswick, New Jersey, United States of America; 4 Universidad de Buenos Aires. Facultad de Ciencias Exactas y Naturales. Departamento de Fisiología y Biología Molecular y Celular. Instituto de Biociencias, Biotecnología y Biología Traslacional (iB3). Buenos Aires, Argentina; International Atomic Energy Agency, AUSTRIA

## Abstract

**Background:**

*Aedes aegypti* (L.) is an urban mosquito, vector of several arboviruses that cause severe diseases in hundreds of million people each year. The resistance to synthetic insecticides developed by *Ae*. *aegypti* populations worldwide has contributed to failures in vector control campaigns, increasing the impact of arbovirus diseases. In this context, plant-derived essential oils with larvicidal activity could be an attractive alternative for vector control. However, the mode of action and the detoxificant response of mosquitoes to plant derived compounds have not been established, impairing the optimization of their use.

**Methods and findings:**

Here we compare gene expression in *Ae*. *aegypti* larvae after 14 hrs of exposure to *Eucalyptus camaldulensis* essential oil with a control group exposed to vehicle (acetone) for the same lapse, by using RNA-Seq. We found differentially expressed genes encoding for cuticle proteins, fatty-acid synthesis, membrane transporters and detoxificant related gene families (i.e. heat shock proteins, cytochromes P450, glutathione transferases, UDP-glycosyltransferases and ABC transporters). Finally, our RNA-Seq and molecular docking results provide evidence pointing to a central involvement of chemosensory proteins in the detoxificant response in mosquitoes.

**Conclusions and significance:**

Our work contributes to the understanding of the physiological response of *Ae*. *aegypti* larvae to an intoxication with a natural toxic distilled from Eucalyptus leafs. The results suggest an involvement of most of the gene families associated to detoxification of xenobiotics in insects. Noteworthy, this work provides important information regarding the implication of chemosensory proteins in the detoxification of a natural larvicide. Understanding the mode of detoxification of Eucalyptus distilled compounds could contribute to their implementation as a tool in mosquito control.

## Author summary

Arboviruses transmitted by *Aedes aegypti* mosquitoes provoke diseases in hundreds of million people yearly. The massive use of neurotoxic insecticides caused the emergence and dispersion of mosquito resistant populations, reducing the available options for vector control. In this context, the implementation of alternative methods is urgent. In order to take full advantage of natural compounds with larvicidal activity, a better understanding of their mode of action and detoxification in *Ae*. *aegypti* larva is necessary. Here, we used transcriptomic and bioinformatic approaches to study the detoxificant response in *Ae*. *aegypti* larvae exposed to an *Eucalyptus camaldulensis*-derived larvicide. Our results revealed a conserved transcriptomic response respect to the intoxication with different xenobiotics. Also, the results suggest an involvement of chemosensory proteins in detoxification. Understanding the mode of detoxification of Eucalyptus distilled compounds could contribute to their use in vector control campaigns.

## Introduction

*Aedes aegypti* is an urban mosquito, vector of several arboviruses that cause diseases such as dengue, chikungunya, zika and yellow fever. Among them, dengue is the most widespread, affecting hundreds of million people every year [1]. Given the distribution of *Ae*. *aegypti* in tropical and sub-tropical areas worldwide, about half of humanity is at risk of contracting a virus transmitted by this vector; the control of mosquito populations is the preferred method for limiting infection rates. With this objective, neurotoxic insecticides such as organophosphates or pyrethroids are used. Insecticide resistance developed by some *Ae*. *aegypti* populations worldwide is an important reason for the failures to control the spreading of arbovirus diseases [2]. Different kinds of insecticides possess different targets in the nervous system. Pyrethroids and DDT, for example, are targeted to the voltage-gated sodium channel, whereas the organophosphates and carbamates act mainly by inhibiting the acetylcholinesterase and neonicotinoids act on nicotinic receptors [3]. Hence, insecticide resistance to different toxics could be caused by mutations in different target genes. Insecticide resistance mechanisms also include changes in expression and activity of detoxifying enzymes [3]. Furthermore, the presence of pollutants in the environment could activate detoxificant mechanisms that confer tolerance to insecticides to mosquitoes [4]. In this context, the implementation of an integrated vector management (IVM) strategy and the development of alternative tools for the control of mosquito populations are urgent [5]. IVM requires the optimal and complementary implementation of all the resources available, in order to minimize the use of neurotoxic insecticides and retard the development of resistance by the use of alternative methods.

Recently, the World Health Organization Vector Control Advisory Group has recommended mosquito traps as a suitable alternative strategy [2]. These traps can be designed to combine attractants for gravid females to lay their eggs, and larvicidal products to kill their offspring. Plant-derived essential oils (EO) with larvicidal activity could be an attractive alternative, given their short environmental persistence, low mammalian toxicity, and cost-effectivity, among other advantages [5]. In particular, EOs distilled from several *Eucalyptus* spp. (EEOs) are considered highly active, with lethal concentrations 50 (LC_50_) ranging from 21 to 95 mg/L, depending on the Eucalyptus species, in strains susceptible to synthetic insecticides [6]. Regarding the mode of action of EOs, effects on GABA, tyramine or octopamine receptors, the inhibition of acetylcholinesterase or genotoxic effects have been proposed with dissimilar evidence [7,8]. However, the exact mode of action of plant derived EOs against *Ae*. *aegypti* larvae has not been established so far.

Insects use metabolic pathways to detoxify xenobiotics through a series of reactions where detoxification enzymes and transporters are involved. Among gene superfamilies related to detoxification, the better studied belong to cytochrome P450s (CYPs), glutathione transferases (GSTs) and carboxyl/cholinesterases (CCEs) [9]. It has been shown that different families and/or members within these superfamilies could more efficiently detoxificate particular xenobiotics and insecticides with respect to others [10]. Although less studied, members of other superfamilies such as ABC transporters, UDP-glycosyltransferases (UGTs) and heat shock proteins (HSPs) were also related with detoxification in insects [11–13]. Also, cuticular modifications help insects to cope with insecticides by means of the impairment of penetration into the body [14,15].

Chemosensory proteins (CSPs) are a family of small soluble proteins present only in arthropods [16]. A role of CSPs in olfaction through the solubilization and transport of hydrophobic odorants has been assumed, even though roles for this protein family in development was also suggested [17]. More recently, direct evidence linking CSPs to detoxification of xenobiotics in insects has been accumulated. Overexpression of CSP members was detected in *Bombyx mori* exposed to avermectin [18], *Plutella xylostella* exposed to pyrethroids [19] and *Bemiscia tabaci* treated with neonicotinoids [20]. In a recent report, Ingham et al [21] demonstrated the direct involvement of a member of the CSP family (named as SAP2 protein) in pyrethroid resistance in *An*. *gambiae*. Furthermore, 4 out of 8 CSPs encoded in the *An*. *gambiae* genome were expressed *in vitro* and binding assays demonstrated their affinity to aromatic compounds [22]. All this evidence strongly indicates that CSPs are involved in xenobiotic detoxification in insects, suggesting that the study of CSPs should be undertaken with this perspective. This will probably contribute to the comprehensive understanding of resistance mechanisms and xenobiotic detoxification.

Metabolic pathways used by mosquitoes to detoxify EOs have been underexplored, even though some results suggested an involvement of CYPs [23,24]. To achieve the full potential of plant-derived EOs for their use in an integrated mosquito management strategy, their mode of action and the metabolic pathways used by mosquitoes to detoxify these compounds should be explored. This information is necessary to achieve a rational design of control strategies which include these natural products, and in the search of tools to improve their efficiency. In this work, we used RNA-Seq as a high throughput approach, in order to identify metabolic pathways involved in the initial response of *Ae*. *aegypti* larvae to an intoxication caused by EEOs. Also, we characterized CSP proteins in *Ae*. *aegypti* genome, and performed a molecular docking analysis to study their putative involvement in the detoxification of *p*-cymene, one of the EEO main toxic components. This combined strategy allowed us to study the response to a natural insecticide in a high-throughput way, revealing putative genes, gene families and pathways involved. We also provide evidence on the involvement of CSPs in detoxification in *Ae*. *aegypti*.

## Methods

### Ethics statement

No human participants, human data or human tissue were used in the present study. Adult mosquitoes were fed on pigeon blood once per week according to a protocol approved by the Institutional Animal Care and Use Committee of CIPEIN (IACUC/CICUAL 1531/13). Insects were housed, cared, fed and handled in accordance with resolution 1047/2005 (Consejo Nacional de Investigaciones Científicas y Técnicas, CONICET, Argentina) regarding the national reference ethical framework for biomedical research with laboratory, farm, and nature collected animals, which is in accordance with the standard procedures of the Office for Laboratory Animal Welfare, Department of Health and Human Services, NIH and the recommendations established by the 2010/63/EU Directive of the European Parliament, related to the protection of animals used for scientific purposes. Biosecurity considerations are in agreement with CONICET resolution 1619/2008, which is in accordance with the WHO Biosecurity Handbook (ISBN 92 4 354 6503).

### Treatment with *Eucalyptus camaldulensis* EO and sample preparation

An insecticide-susceptible strain of *Ae*. *aegypti* (Rockefeller strain, Venezuela) was used in the bioassays. The colony has been kept in the laboratory since 1996, free of exposure to pathogens, insecticides, or repellents, at 25–30°C, 80–90% of relative humidity, and a L12/D12 photoperiod [25]. We selected this strain for the analysis, instead of a wild population, given that we are able to track its previous history of exposure to toxics and its status of susceptibility. All larval instars were fed on a mixture of rabbit pellets and yeast in a 3:1 proportion.

The larvicidal bioassay was performed with 1 mL of the *E*. *camaldulensis* EO diluted in pure acetone (Merck, Darmstadt, Germany), which was added to 224 mL of dechlorinated water in a 500-mL plastic jar, to a final concentration of 26.75 mg/L, which is the LC_50_ for this EO (see extraction and analysis procedures of *E*. *camaldulensis* EO in [25]. The mixture was shaken slightly to ensure a homogeneous solution. Then, 20 late third or early fourth instar *Ae*. *aegypti* larvae were placed in 25 mL of dechlorinated water and transferred to that jar. For the control group, 20 larvae were introduced in a jar containing 1 mL pure acetone in 249 mL of dechlorinated water. No food was offered to the larvae during the exposure time given that, if the treatment affects the feeding behavior of the larvae, this would differentially affect the expression of genes in both experimental groups. Hence, we would not be able to differentiate those changes in gene expression due to the intoxication itself from those due to differences in feeding condition/nutritional state. The bioassays were conducted in a 27±2°C regulated chamber, with 80–90% relative humidity and a 12:12 hrs photoperiod [25]. After 14 hrs of exposure, surviving larvae from both experimental groups were collected in microtubes containing Trizol (Ambion, Sao Paulo, Brazil) (n = 8-10/group); this reagent was also used for total RNA extraction, according to the manufacturer’s instructions. We chose a 14 hrs period of exposure in order to allow differences in gene expression to reach a maximum, based on previous results on *Ae*. *aegypti* larvae intoxication [26]. Larvae were considered dead following the method previously reported [27]. The bioassay was repeated four independent times in order to obtain four independent biological replicates for each experimental group.

### RNA sequencing and bioinformatic analysis

Library construction and high-throughput sequencing services were hired at Novogene Corporation Inc. (Sacramento, USA). A total of 8 cDNA libraries (4 per experimental condition) were constructed using the NEBNext Ultra RNA Library Prep Kit (New England Biolabs) with an insert length of 250–300 base pairs (bp). The libraries were sequenced using Illumina NovaSeq (paired-end reads with 150 bp length) with a sequencing depth of at least 26.9 million per library. The raw sequence dataset is available with the NCBI-SRA Bioproject number PRJNA671513.

The FASTQC tool [28] was used to analyze the presence of Illumina sequencing adapters and the read quality. After, Illumina adapters and those bases from 5’ and 3’ ends with Phred quality scores lower than 5 (TRAILING: 5 and LEADING: 5 parameters) were removed from the reads using Trimmomatic v0.32 in the paired-end mode [29]. Besides, the SLIDING-WINDOW parameter was set as 4:15 and only reads longer than 50 bp were maintained (MINLEN parameter = 50). The last version of the *Ae*. *aegypti* genome (Liverpool AGWG strain with the assembly AaegL5.0, uploaded on June 2017) was downloaded from VectorBase [30] with its corresponding General Feature Format (GFF) file, a tab-delimited text file that describes the genomic features (annotation AaegL5.2, uploaded 24th April 2019). STAR v.2.6.0 [31] was used to index the genome file and to map the trimmed reads with default parameters. The *htseq-count* command (with parameters *-t* exon *-i* Parent *-r* name and *-s* no) of HTSeq v.0.11.1 [32] was used to report the counts of the mapped paired-end reads from multiple index BAM files at specific intervals.

Gene expression analysis was carried out using edgeR package v3.6.8 [33] and the count data generated by HTSeq were used as input in R-studio. Read counts were initially normalized using *calcNormFactors* function, which implements the Trimmed Mean of M-values (TMM) method. Following, the *estimateDisp* function that calculates gene-specific biological variation (*tagwise*) based on the empirical Bayes method was applied. Afterwards, normalized counts were analyzed using the GLM approach and the quasi-likelihood F-test. First, the *glmQLFit* function was used to fit read counts to a quasi-likelihood negative binomial generalized log-linear model and, afterwards the *glmTreat* function was used to test for differential expression relative to a minimum required fold-change threshold = 2. Second, genes with low expression and high variation were filtered using HSTFilter package v. 1.32.0 [34]. Finally, we used the *topTags* function to rank and extract the differentially expressed genes according to their False Discovery Rate (FDR) values. Those genes with an FDR<0.05 were considered as differentially expressed genes (DEGs) between control and treated groups. We extracted the normalized Counts Per Million (CPM) values of the DEGs from the detoxification related protein families. A heatmap was generated using pheatmap v.1.0.12; this package calculates Z-scores (subtracting the mean and then dividing by the standard deviation) for each gene and plotted instead of the normalized CPMs.

The GO-terms of the *Ae*. *aegypti* predicted proteins were obtained from VectorBase [30] using the BioMart tool. The enrichment analysis was carried out with ermineR package using the Gene Score Resampling method (GSR) [35] and the absolute log fold change values for each gene to produce a score rank. In order to have a global analysis, complementary to that of the DEG set, all the genes in the database were considered for this analysis, along with their corresponding logFc as a continuous variable. A total of 200.000 interactions were performed. With this strategy, GO-terms that were enriched along the whole dataset were detected. For details on the strategy used see https://erminej.msl.ubc.ca/help/tutorials/running-an-analysis-resampling/.

In parallel, DEGs were mapped to the KEGG PATHWAY database and Fisher’s exact test followed by Benjamini and Hochberg FDR correction method was used to identify significantly enriched pathways (FDR<0.05) using KOBAS 3.0 server [36]. The pipelines used to perform the differential gene expression analysis (with EdgeR) and the GO-enrichment analysis (with ermineR) are available at: https://github.com/josmantorres/Differential-Gene-Expression-and-Enrichment-Analysis-pipeline.git

According to previous literature [37], we operationally defined a gene cluster when N genes belonging to the same gene family are arranged within a genomic region having fewer than N—1 genes that don’t belong to this family.

### Identification, enrichment and clustering analysis of detoxificant-related gene families

The PFAM domains were used in the HMMER tool (Biosequence analysis using profile hidden Markov models) and as queries in tBLASTn searches [38] on the AaegL5.2 gene set (published on 24 Apr 2019). We used the following PFAM domain alignments as queries: PF00011 (HSP20); PF00012 (HSP70); PF00005 (ABC-transporters); PF00067 (CYP450); PF02798, PF00043 (GSTs); PF00201 (UGT) and PF03392 (CSP). The microsomal GSTs were identified using the orthologues from *Drosophila melanogaster* as queries. Those transcripts annotated as “pseudogenes” were not considered.

We used *cpm (y*, *prior*.*count = 10*, *log = TRUE)* function in e dgeR to generate a matrix of log2 CPM that was used to produce heatmaps, including all the members from each protein family, by means of gplot package v.3.1.1 in R-studio. Dendrograms were plotted with hierarchical clustering among genes based on Euclidean distances and complete linkage method for clustering. A g ene set enrichment analysis was conducted with each one of the detoxification-related gene families using the “Category” package (v.2.58.0) with the hyperg function, which performs a hypergeometric test for over- or under-representation of significant ‘genes’ amongst those assayed in a universe of genes. For each gene family, two lists were generated based on their PFAM domains: IDs of family members identified as differentially expressed (named significant); and IDs of family members identified in the entire genome (named assayed). Both lists were compared among them and with a third one containing the IDs of the entire genome with at least one PFAM domain (11.931 genes). The pipeline used for the hypergeometric test was also included in the GitHub link mentioned above.

### Characterization of Chemosensory Proteins in *Ae*. *aegypti*

Analysis of CSPs was performed for the detection of a signal peptide (predicted by SignalP 5.0 [39]), the four characteristic cysteine domains and a secondary structure including six α-helices (predicted by PSIPRED [40]. For phylogenetic analysis, sequences were aligned in Multiple Alignment using Fast Fourier Transform (MAFFT) software version 7.0 (using G-INS-i strategy with—unalignlevel 0.1—leavegappyregion—ep 0.12—maxiterate 1000) and the resulting alignment was trimmed with trimAl v1.2 (using gap threshold = 0.3, [41]). Finally, maximum likelihood trees were built in IQ-TREE [42] and edited with iTol (https://itol.embl.de/). Branch support was determined using the approximate Likelihood Ratio Test (aLRT). Non-parametric branch support was based on the Shimodaira-Hasegawa-like (SH) procedure. CSP sequences from *An*. *gambie* [43] were included in the phylogenetic analysis in order to infer homologies.

### Molecular modelling

CSP sequences were used to generate structural models using Modeller 9.25 [44]. A combination of multiple structures deposited in RSCB PDB [45] (1k19,1kx8,1n8u,1n8v,2gvs,2jnt) were used as templates, following the procedure indicated in modeller 9.25 manual for advanced modelling. A total of 100 models were built for each CSP and the one with the best (lower) Discrete Optimized Protein Energy (DOPE) score was selected.

### Molecular docking

Each CSP structure was considered as a receptor in independent runs, using the modeled structure described in the previous section. *p*-cymene ligand structures were obtained from Drugbank 5.0 [46]. Previous to docking runs, receptor and ligand were prepared using the prepare_ligand.py and prepare_receptor.py scripts from MGLtools suite [47]. Docking runs were performed using Autodock VINA 1.1.2 [48], with a box that fully covers the receptor, under default parameters and producing 10 modes *per* complex. For each CSP, the lowest (best) and average binding energy is reported, together with the root-mean-square deviation (RMSD) for the different binding modes.

## Results and discussion

### Sequencing and mapping metrics

RNA-Seq analysis generated more than 245 million reads with an average of 30.68 million reads *per* experimental replica. More than 97.5% of raw reads were retained after the trimming and filtering processes. High quality reads were further mapped to *Ae*. *aegypti* genome with an average of 24.65 million reads *per* replica (which represents 82.4% of the trimmed reads) (S1 Table). Principal component analysis showed that samples under the same treatment were grouped (control *vs*. EEO treated; S1 Fig).

### Differential expression analysis after treatment with *E*. *camaldulensis* EO

Differential transcription analysis was performed on 11.151 transcripts (78% of the total predicted transcripts). A total of 239 genes (2.1% of the analyzed transcripts) were found differentially transcribed with an absolute fold-change >2 and an FDR<0.05 in the EEO treated group. These DEGs included 177 transcripts over-transcribed and 62 under-transcribed (see the complete list of DEGs in S2 Table and S2 Fig for a volcano plot).

Forty-two of the DEGs (17.6%) belong to gene families previously associated with detoxification in insects (13 HSPs, 9 CYPs, 6 UGTs, 5 CSPs, 5 GSTs and 4 ABC transporters).

**Fig 1:**
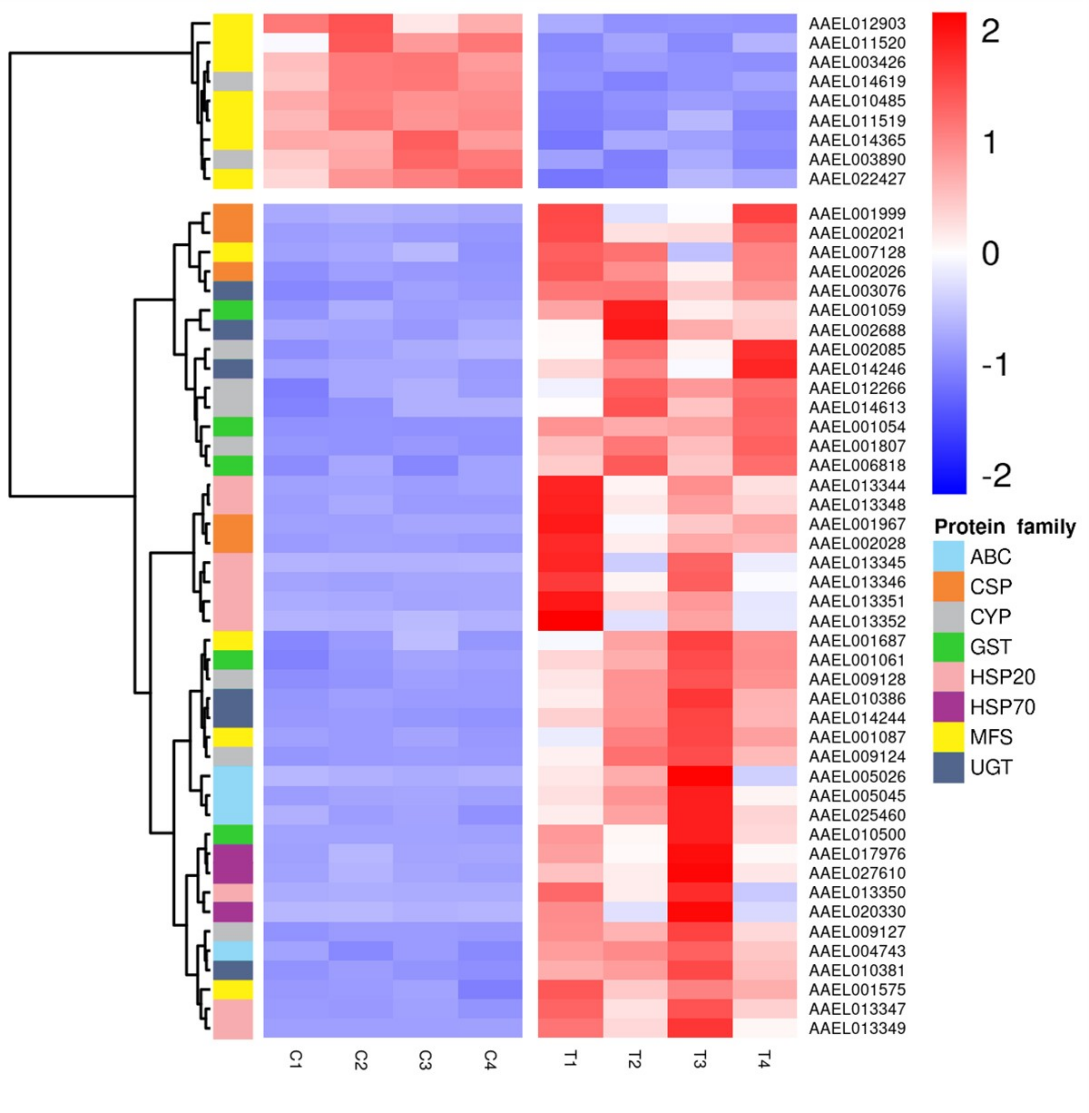
Effect of EEO treatment on the transcription of differentially expressed genes related to xenobiotic detoxification families and major facilitator superfamily. Heatmap was prepared using pheatmap that calculated z-score for each gene and plotted by means of a color scale, in which blue/red represent lowest/highest expression. Genes are identified by their VectorBase ID. Dendrogram was plotted with hierarchical clustering among samples and genes based on Euclidean distances and Ward method for clustering. C: control samples. T: treated samples.

From these, only 2 CYPs were underexpressed (AAEL003890 and AAEL014619/CYP9J22), the remaining detoxificative-related transcripts were overexpressed after EEO treatment (Fig 1). None of the members of CCEs, a superfamily related to xenobiotic detoxification in insects [49,50], was present in the DEG set (S2 Table). Conversely, previous transcriptomic studies demonstrated differential expression of CCEs in *Ae*. *aegypti* larvae exposed to the carbamate propoxur and the neonicotinoid imidacloprid, but not to the pyrethroid permethrin [4]. Besides, larvae from a population resistant to propoxur [51] and female adults resistant to the pyrethroid deltamethrin [52] presented differentially expressed CCEs members. On the other hand, *Aedes albopictus* larvae resistant to the organophospate larvicide temephos also presented an overexpression of CCEs when compared to a susceptible population [53]. Altogether, our results and previous data suggest that the transcriptional regulation of CCEs in response to an intoxication could be particular for different kinds of xenobiotics.

Besides those genes directly involved in detoxification processes, we identified DEGs belonging to families that could have an important role in the defense against toxic xenobiotics, such as fatty acid synthesis related genes and cuticular proteins (S2 Table). The involvement of cuticular proteins and synthesis of cuticular lipids has been related to insecticide resistance as much in mosquitoes as in other species [14,15]. Overexpression of many genes encoding for cuticular proteins has been reported in *Ae*. *aegypti* larvae in response to propoxur, imidacloprid and permethrin, whereas the transcription of genes involved in lipid metabolism was detected in response to propoxur and imidacloprid [4]. Interestingly, we found that 19 (7%) of the DEGs encode proteins related to lipid metabolism (17) or cuticle (2) (S2 Table). From DEGs involved in lipid metabolism, 12 are overexpressed and 5 under-expressed in larvae exposed to EEO (S2 Table). Most of the overexpressed genes have a role in fatty acids biosynthesis (i.e. elongase, Acetyl-CoA synthase, AMP dependent ligases), whereas 3 out of 5 of the under-expressed are lipases, suggesting a net augment in fatty acid concentration. Besides, two cuticular-related genes were found differentially expressed; one cuticular protein was overexpressed, and a chitinase was under-expressed. Altogether, the results indicate that the cuticle reinforcement could be a mechanism of *Ae*. *aegypti* larvae to cope with a sustained exposure to EEO.

Hexamerins are abundant proteins in hemolymph, which have storage and transport roles, and were previously associated with resistance to insecticides in mosquitoes, in particular to pyrethroids [11,51], even though the exposure to synthetic insecticides or other xenobiotics did not modulate the expression of hexamerins [4]. In agreement, none of the transcripts modulated by the exposure to EEO belongs to the hexamerin family (S2 Table). These results suggest that, in spite of the probable role of hexamerins in insecticide resistance, their basal expression is not affected during the detoxificant response.

Major facilitator superfamily (MFS) members are membrane transports phylogenetically related [54]. They are involved in the transport of solutes through cell membranes. Opposite to ABC transporters that use ATP, MFS transport substrates in favor of an electrochemical gradient [54]. A role of MFS in the transport of toxic substances in bacteria and fungi has been demonstrated [55–57]. In our work, we found that 10 DEGs were MFS transporters. Besides, other 3 genes are solute transporters independent of ATP, but not presenting a MFS domain (AAEL005353, AAEL007458, and AAEL013109; S2 Table). Eight of these transporter genes were downregulated by the EEO treatment (Fig 1 and S2 Table; FDR<0.05). The tendency to downregulate the expression of genes involved in the transport of solutes in favor of their electrochemical gradient could help in the preservation of cells from intoxication. Interestingly, MFS expression modulation has been previously observed in arthropods exposed to toxins derived from plants [58,59], but not in *Ae*. *aegypti* larvae exposed to pollutants or synthetic insecticides [4].

Other DEGs encode mostly metabolic enzymes such as alcohol-dehydrogenases, hydrolases, peptidases, kinases/phosphatases, oxidases, reductases and transferases (S2 Table). Finally, the expression of one caspase, one odorant binding protein and two proteins induced by juvenile hormone were detected as upregulated (S2 Table).

**Fig 2:**
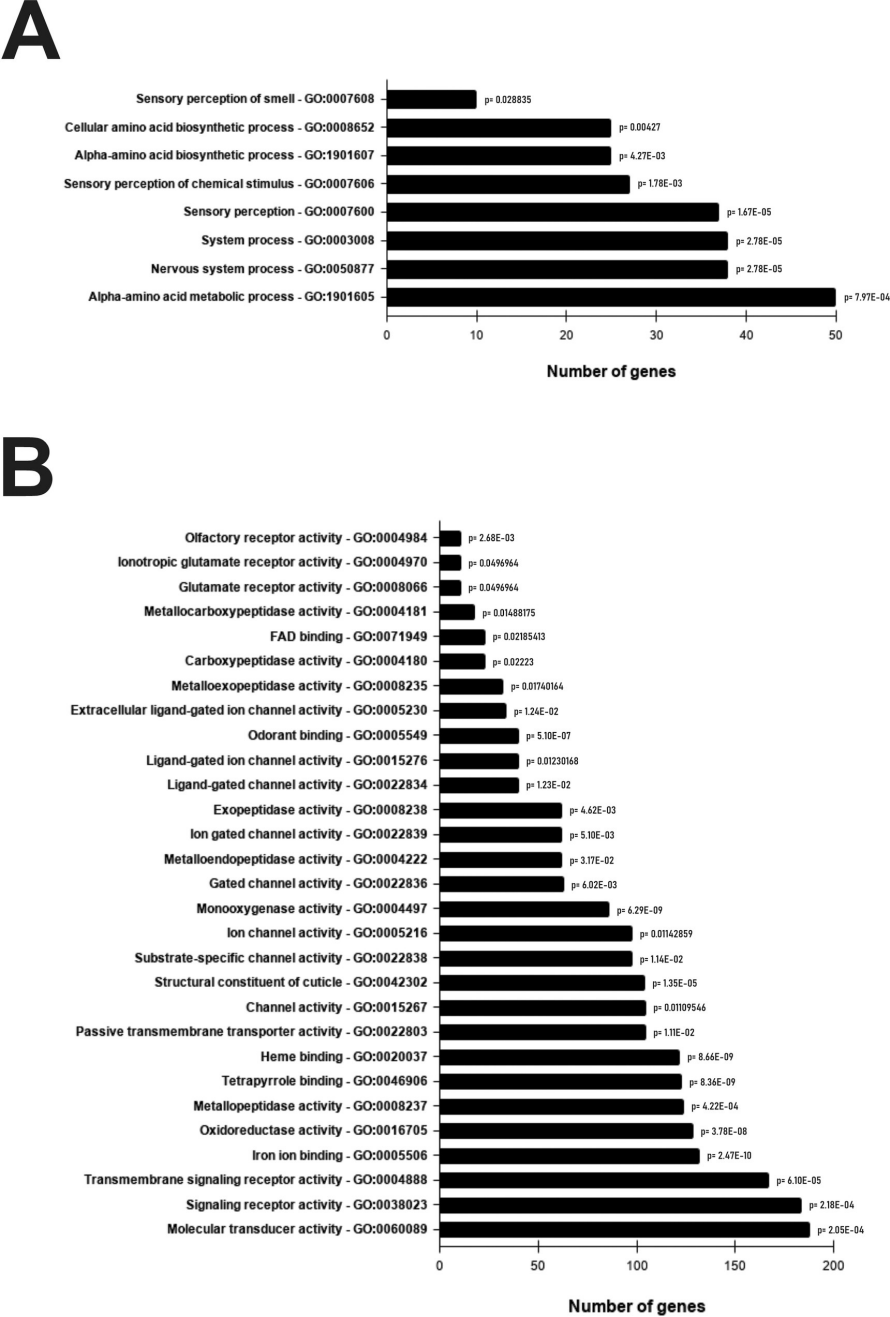
GO enrichment analysis of the RNA-Seq data. **A.** Biological process. **B.** Molecular Function. Functional gene sets were defined using the Gene Ontology (GO) annotations of the *Aedes aegypti* genome (AaegL5.3 version) in VectorBase. Gene Score Resampling (GSR) method was applied and the reported IDs correspond to the significantly enriched GO-terms (FDR<0.05).

Consistently with the DEG set, the GO-enrichment analysis revealed that biological processes related to biosynthesis and metabolism of alpha-amino acids, as well as sensory perception, were significantly enriched in the treated samples (Fig 2A). Considering the GO molecular function terms (Fig 2B), several activities were affected after the treatment: 1) detoxification, including iron, FAD and heme binding together with several metabolic enzymes (peptidase, reductases and monooxygenases); 2) odorant binding and sensory perception; 3) signaling and transduction and 4) structural constituents of the cuticle. The results of the KEGG enrichment analysis were similar to those generated by GO-term analysis, reinforcing the effect of the EEO at the metabolic level. For the complete list of KEGG enriched terms (FDR<0.05) see S3 Table).

### Families potentially involved in detoxification

**Fig 3:**
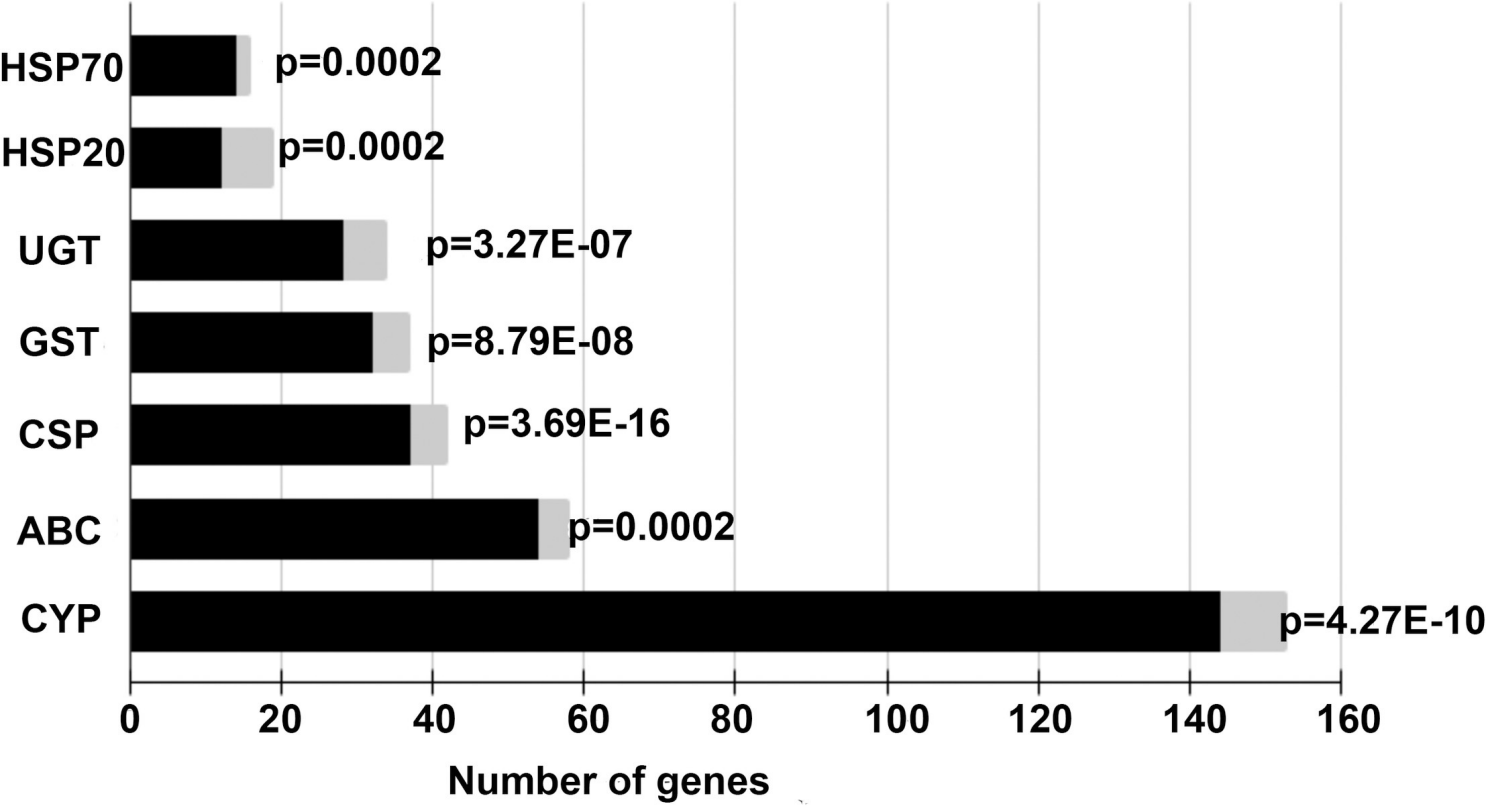
Hypergeometric test results for enrichment analysis of gene families potentially involved in detoxification. Horizontal bars indicate total gene numbers. Black bars indicate the number of genes whose expression was unaltered after EEO treatment and gray bars indicate the number of genes differentially expressed in each gene family. P-values for each family are indicated. HSP20: Heat shock proteins 20; HSP70: Heat shock proteins 70; UDP: UDP glycosyltransferases; GST: Glutathione transferases; CSP: Chemosensory proteins; ABC: ABC transporters; CYP: Cytochromes P450.

A hypergeometric test was performed in order to evaluate the enrichment of HSP20, HSP70, CYP, UGT, GST, ABC-transporters and CSP gene families in the DEG set. All these gene families were over-represented and significantl enriched in this set (p<0.001; Fig 3).

### Heat shock proteins

HSP expression is induced by a variety of stresses in insects, including heat and insecticide treatment [26,60]. We found that 12 DEGs are HSPs, all of them over-expressed in mosquito larvae treated with EEO. Nine of the HSP upregulated genes belong to the HSP20 (α-crystalline) class, whereas 3 belong to the HSP70 class. Also, one HSP20-like_chaperone (AAEL013314), which does not contain the HSP20 domain, was upregulated (FDR<0.05) (S2 Table).

The involvement of HSP20 in insecticide resistance in *An*. *gambiae* has been recently demonstrated [11]. Furthermore, the expression of HSP20 members is modulated in *An*. *gambiae* in response to treatment with a pyrethroid [11], but previous transcriptomic analyses did not report changes in the expression of HSPs in *Ae*. *aegypti* larvae in response to treatment with synthetic insecticides nor in resistant populations [4,51]. We detected 17 putative HSP20 sequences in *Ae*. *aegypti*transcript dataset; all these transcripts are located in chromosome 2, conforming a gene cluster that spans <200,000 bp in the genome. Besides those 9 genes that reached FDR<0.05, most of the HSP20 transcripts tended to be upregulated in insects treated with EEO (see heat maps in S3 Fig), indicating that this family could be involved in the response to EEO in *Ae*. *aegypti*. Also, the disposition of HSP20 genes in a cluster and its expression pattern could indicate a transcriptional co-regulation. In agreement, transcriptional co-regulation of HSP20 members was suggested in *An*. *gambiae* [11].

The expression of most HSP70, which are overexpressed in response to heat stress in mosquitoes [61], tended to be upregulated during exposure to EEO, 3 of them reached statistical significance (FDR<0.05) (S3B Fig).

### Cytochromes P450

From the 7 overexpressed CYPs, 3 belong to the CYP6 family, 2 to CYP9 family and 2 to CYP4 family (S2 Table). From these, AAEL001807/CYP9M9 was also observed overexpressed in response to both imidacloprid and propoxur [4], and in larvae from populations resistant to these two insecticides [51]. This could point to a role of CYP9M9 in the detoxificant response to a wide range of toxics.

The 3 CYP6 that were overexpressed with exposition to EEO (AAEL009124/CYP6N12, AAEL009127/CYP6M11 and AAEL009128/CYP6M6) were located in the same gene cluster in the genome, suggesting a co-regulation and/or a comparable role. Besides, one underexpressed CYP (AAEL014619/CYP9J22) belongs to CYP9 family, which was previously associated with detoxification in insects, particularly in *Ae*. *aegypti* [51,62]. Several CYP genes tended to be either underexpressed or overexpressed after exposure to EEO and most of these genes were classified in CYP9 or CYP6 families. Treatment with permethrin, which belongs to the pyrethroid family widely used as an adulticide for mosquitoes [2], did not modify the expression of CYPs in *Ae*. *aegypti* larvae [4], and the expression of three CYP6 and one CYP12 was underexpressed in larvae from a permethrin-resistant population [51]. Besides, in the larvae resistant to imidacloprid and in those resistant to propoxur, the expression of two genes encoding CYP6 (AAEL009131 and AAEL 014893) and one gene encoding a CYP9 (AAEL001807) were found upregulated [51]. Altogether, the results suggest that the members of CYP family involved in detoxification could differ with dependence of the toxic stimulus applied to *Ae*. *aegypti* larvae.

### UDP-glycosyltransferases

Enzymes belonging to UDP-glycosyltransferase (UGT) superfamily catalyze glucosidation and transfer of glycosyl from UDP-glycosyl donor to a lipophilic molecule [13]. This superfamily has been involved in insect resistance to both plant allelochemicals and insecticides [63,64]. In our study, we found 6 overexpressed UGT encoding genes (S2 Table). Besides several other members of this superfamily tended to be overexpressed in the larvae treated with EEO (S3D Fig). From this gene family, AAEL003102 was overexpressed in untreated larvae from resistant populations to imidacloprid and propoxur [51], even though the transcription of this gene was not affected by treatment with EEO.

#### Glutathione transferases

GSTs are involved in insecticide detoxification in diptera, particularly those belonging to Delta and Epsilon families [65]. After 14 hrs of exposure to EEO, 5 genes belonging to GST superfamily were differentially overexpressed (S3E Fig). From these, 3 belong to the Delta family (AAEL001054/GSTD4, AAEL001059/GSTD3, and AAEL001061/GSTD1) and were located in a genome cluster in chromosome 1. In particular, GSTD4 expression was induced by different synthetic xenobiotics [5]; the expression of a close orthologue of this enzyme was upregulated in larvae of *Ae*. *albopictus* resistant to temephos [52]. The remaining differentially expressed GSTs (FDR<0.05) were AAEL010500/GSTX2 and AAEL006818. The former is conserved among mosquito species [66]; its expression was induced in response to propoxur [50], and its orthologue in *Ae*. *albopictus* was elevated in response to temephos [52]. AAEL006818 is a microsomal GST; a class of GSTs that was not previously involved in detoxification response in insects.

#### ABC transporters

Four ABC transporter genes were overexpressed under treatment with EEO, all of them belonging to ABCC subfamily [67] (S3F Fig); both AAEL005026 and AAEL005045 were grouped in the same gene cluster in chromosome 2. ABCC subfamily has been previously associated to multidrug resistance and insecticide detoxification [67]. One of the differentially overexpressed ABCC (AAEL025460, previously named AAEL005937) has been associated with pyrethroid resistance in *Ae*. *aegypti* [62]. Treatments with imidacloprid or propoxur modulated the expression of members of ABC transporters family [4] but the particular transcripts affected did not overlap among the response to different toxics.

### Chemosensory proteins

Forty-two transcripts encoding CSPs were detected in *Ae*. *aegypti* genome, 5 out of these genes were overexpressed in *Ae*. *aegypti* larvae treated with EEO (S3G Fig; FDR<0.05; AAEL001967, AAEL001999, AAEL002021, AAEL002026, and AAEL002028).

**Fig 4:**
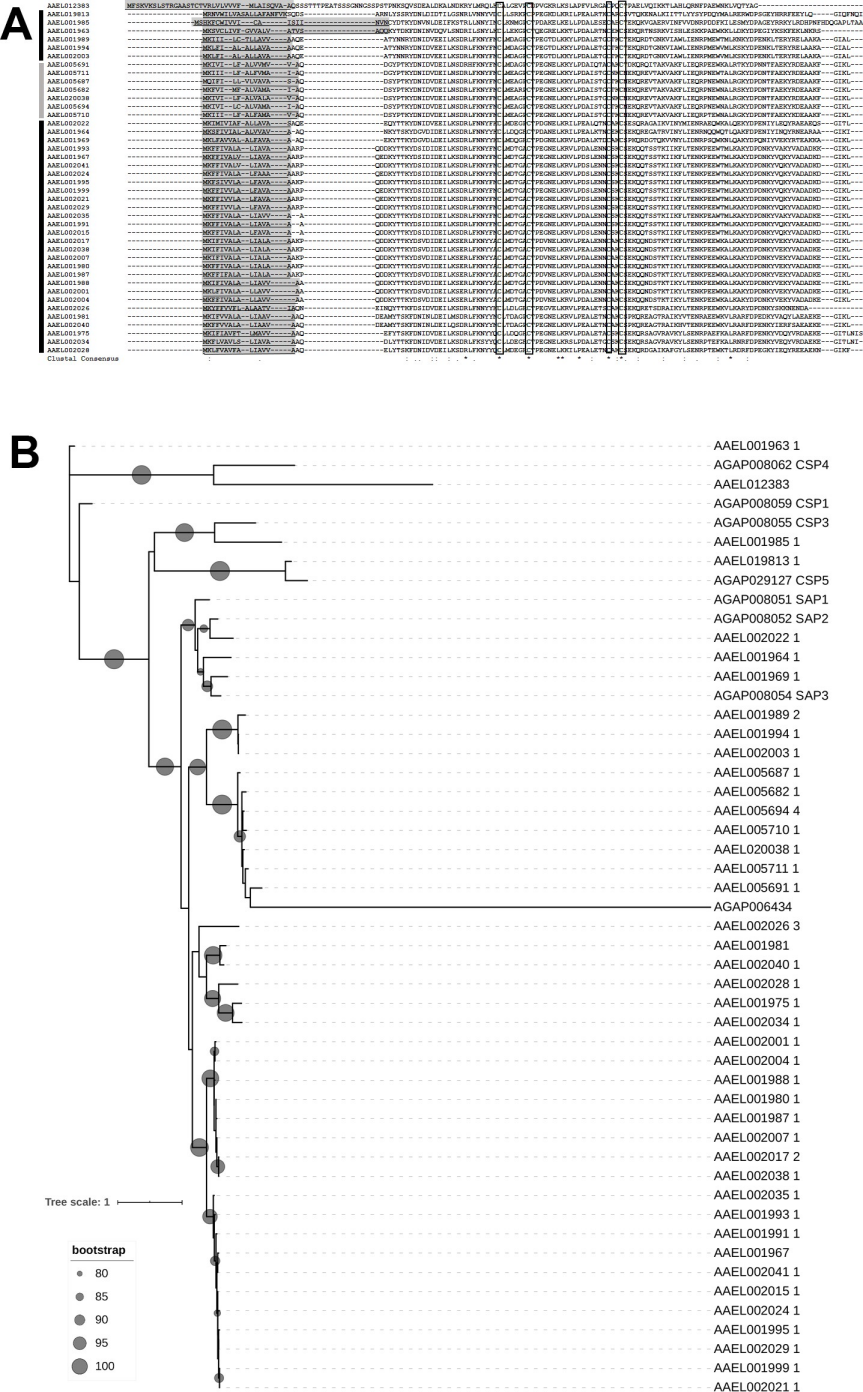
A. Multiple sequence alignment of chemosensory proteins identified in *Ae*. *aegypti* genome. Predicted signal peptide sequences are indicated underlined and with a light-gray shadow. Conserved cysteine residues are boxed. For clarity reasons, only the conserved region of the larger sequences (AAEL001985 and AAEL019813) are shown. In the last line of each alignment, an asterisk indicates a fully conserved residue, a colon indicates a conservative substitution with strongly similar properties, and a period indicates a semiconserved substitution with weakly similar properties. Black bar in the left indicates sequences located in chromosome 2 cluster; gray bar indicates sequences located in chromosome 3 cluster. B. Phylogenetic analysis of *Ae*. *aegypti* and *An*. *gambiae* chemosensory proteins constructed on IQ-Tree using the LG+I+G4 (Best-fit model according to Bayesian Information Criterion) as a model of amino-acid substitution and it is based on 1,000 replicates. The branch support values were estimated using the approximate Likelihood Ratio Test based on the Shimodaira-Hasegawa (aLRT-SH) procedure.

All of the CSP members found in *Ae*. *aegypti* genome presented the hallmarks of this protein family: the signal peptide, a pattern of 4 cysteines and 6 α-helical segments (Fig 4A). However, we found that the members of the CSP family have been annotated in the *Ae*. *aegypti* genome as “protein serine/threonine kinase” (www.vectobase.org). CSPs in *Ae*. *aegypti* genome are disposed in two clusters, one in chromosome 2 containing 34 genes, and a second one in chromosome 3 counting 7 genes (Fig 4A). The only CSP encoding gene outside of these clusters was AAEL012383, located in chromosome 3. The organization in clusters is a usual finding in CSP families [68], suggesting that large families are originated by gene duplications. The conservation of clusters in different species suggests an evolutive pressure to maintain this organization in the genome [68], and could point to a coordinated regulation of the gene expression. Interestingly, all the overexpressed CSPs are located in the chromosome 2 cluster. Other CSP encoded in this cluster (AAEL002024) also tended to be overexpressed (FDR = 0.077).

A bibliographic survey revealed that the expression of members of the CSP family (previously misinterpreted in the genome automatic annotation as “protein serine/threonine kinase”) has been also significantly modulated in *Ae*. *aegypti* populations resistant to different kinds of insecticides [50]. This kind of modulation also occurred in response to xenobiotics or synthetic insecticides[5]. We observed that the CSPs modulated both by xenobiotics and in resistant populations of *Ae*. *aegypti* were all located in the chromosome 2 cluster, reinforcing the evidence that suggests an involvement of this group of CSPs in detoxification. Besides, AAEL002028 was significantly upregulated both with EEO and with imidacloprid, whereas AAEL002021 was upregulated both with EEO and propoxur. Several m embers of the CSP family were found to be differentially regulated in *Ae*. *aegypti* larvae resistant to synthetic insecticides [51], but none of them overlapped with those CSPs overexpressed during the treatment with EEO. Furthermore, treatments with imidacloprid or propoxur modulated the expression of different sets of CSPs (6 transcripts were modulated only by imidacloprid; 6 transcripts were modulated only by propoxur and 3 transcripts overlapped in both responses) [4]. These results, in agreement to previous observations in *An*. *gambiae* [22], point to a specificity of different CSPs for dealing with different xenobiotics.

The number of CSP paralogues detected in *Ae*. *aegypti* (42) is much bigger than the reported for most mosquito genomes (8 in *An*. *gambiae*, 27 in *Culex quinquefasciatus*) [42,68,69], even though *Ae*. *albopictus* genome encodes 83 CSPs [69]. We performed a phylogenetic analysis to assign possible orthologues between *Ae*. *aegypti* and *An*. *gambiae* CSPs (Fig 4B). We observed orthologies between AAEL001963 and AgamCSP1, AAEL001985 and AgamCSP3, AAEL012383 and AgamCSP4, AAEL019813 and AgamCSP5. Besides, SAP1, SAP2 and SAP3 were grouped with most of the CSP proteins located in chromosome 2 cluster in the *Ae*. *aegypti* genome. Hence, the phylogenetic analysis indicates that CSPs clustered in chromosome 2 are orthologues of SAP proteins from *An*. *gambiae*, which were demonstrated to have a role in detoxification [22]. In parallel to the pattern of expression modulation with xenobiotics (see above), a detoxificant role of *Ae*. *aegypti* CSP clustered in chromosome 2 can be suggested as a suitable hypothesis for further physiological experiments.

The expansion observed in the CSP complement and the probable involvement of this gene family in detoxification suggest that *Ae*. *aegypti* could be especially versatile to deal with different toxic molecules and, as a consequence, to develop resistance to a wide spectrum of insecticides.

**Fig 5:**
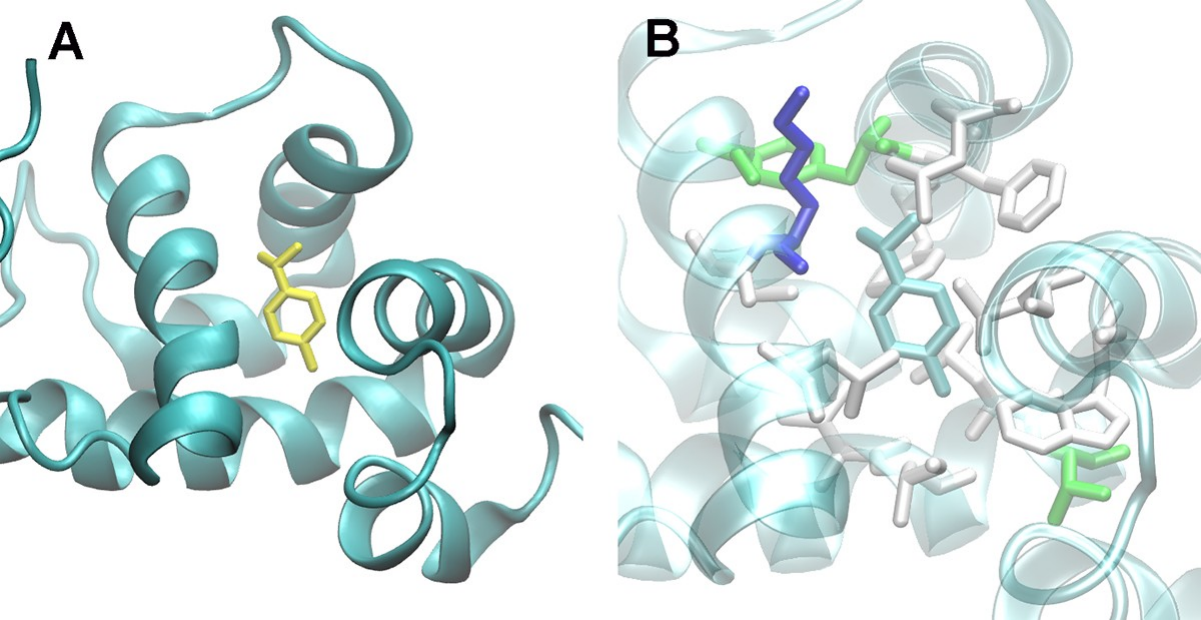
Docking of *p*-cymene inside a CSP. A. Best binding mode of *p*-cymene (yellow) within AAEL001999 protein (ribbon representation). B. Zoom of the binding cavity, highlighting as solid colored sticks all amino acid sidechains within 5 Å of the ligand.

The main components of *E*. *camaldulensis* EO are 1,8-cineole, *p*-cymene and β-phellandrene [26]. According to previous results, *p*-cymene seems to be centrally implicated in toxicity against *Ae*. *aegypti* larvae [26]. Bioassays on larvae exposed to 40 parts *per* million of pure *p*-cymene exhibited a mortality rate of 100%, and there exists a significant correlation between larval mortality and the concentration of *p*-cymene in the EEO [26]. To further study the probable role of CSPs in EEO detoxification, we modeled the docking of *p*-cymene in all the members of the CSP family in *Ae*. *aegypti*. We found a favorable energy for docking in all the *Ae*. *aegypti* CSPs, going from -7.6 (AAEL001993) to -4.7 (AAEL002041) kcal/mol (Table 1). Two of the CSPs that are significantly overexpressed after EEO exposition (AAEL001999 and AAEL002021) also presented a highly favorable interaction energy (-7.3 kcal/mol for both). Fig 5 reports the best mode of AAEL001999 interaction with *p*-cymene found, where the aromatic/hydrophobic amino acids Phe42, Phe46, Leu49, Leu62, Leu66, Ile88, Ile89, Leu92, Trp100 and Leu103 are evidenced as defining the binding cavity. This cavity is bigger than the ligand and can hold it in two orientations (not shown). Given the size and hydrophobic nature of the cavity it could also bind other hydrophobic molecules of similar size. Furthermore, the nature of the residues surrounding the cavity in different CSPs could confer specificity/preference for different ligands. Altogether, binding analysis suggests a role of CSPs as detoxicants, with particular involvement of AAEL001999 and AAEL002021 for detoxification of *p*-cymene, a central component of EEOs. The overexpression of these genes in the presence of *p*-cymene could help to sequester the toxic molecules, reducing in this way its effects on physiology and survival of larvae.

**Table 1:** Docking of *Ae*. *aegypti* CSPs with *p*-cymene.

Accesion	Affinity (kcal/mol)	Avg_Affinity	rmsd_lb	rmsd_lb
AAEL001993	-7.6	-5.86	5.76	7.7
AAEL001999	-7.3	-5.95	5.43	7.44
AAEL002021	-7.3	-6.1	3.78	5.98
AAEL005682	-6.9	-5.52	6.71	8.12
AAEL001980	-6.8	-4.75	10.46	12.3
AAEL002024	-6.7	-6.21	3.97	5.71
AAEL001985	-6.6	-5.46	4.04	5.83
AAEL001987	-6.5	-5.05	7.37	8.83
AAEL002001	-6.5	-5.89	7.51	9.13
AAEL002026	-6.2	-5.22	2.07	4.32
AAEL019813	-6.1	-5.04	7.43	9.09
AAEL002028	-6	-5.17	13.38	15.3
AAEL012383	-6	-5.72	4.16	6.12
AAEL001989	-5.9	-4.88	8.89	10.84
AAEL002040	-5.9	-5.05	4.95	6.95
AAEL001988	-5.8	-5.22	3.51	5.67
AAEL001995	-5.7	-4.79	7.04	8.9
AAEL002007	-5.7	-4.89	6.18	7.91
AAEL002017	-5.7	-4.95	5.59	7.55
AAEL002038	-5.7	-5.05	4.59	6.54
AAEL001969	-5.6	-4.85	3.83	5.78
AAEL001981	-5.6	-4.84	11.18	12.68
AAEL002035	-5.6	-5.02	11.03	13.08
AAEL005687	-5.6	-4.9	2.81	5
AAEL005710	-5.6	-5.07	4.48	6.2
AAEL005711	-5.6	-4.99	1.31	3.51
AAEL002003	-5.4	-4.81	5.18	6.64
AAEL002004	-5.4	-4.79	9.33	11.03
AAEL002015	-5.4	-4.88	2.37	4.7
AAEL002034	-5.4	-4.82	10.86	11.97
AAEL020038	-5.4	-4.92	2.45	4.34
AAEL001963	-5.3	-4.7	3.53	5.48
AAEL001975	-5.3	-4.85	10.48	11.81
AAEL001994	-5.3	-4.64	9.26	10.53
AAEL002029	-5.3	-4.86	3.96	5.61
AAEL001964	-5.2	-4.73	6.16	7.96
AAEL001967	-5.2	-4.84	7.39	9.31
AAEL005691	-5.2	-4.94	2.18	4.1
AAEL001991	-5.1	-4.59	7.36	8.67
AAEL005694	-5	-4.62	7.4	8.87
AAEL002022	-4.9	-4.48	7.11	9.19
AAEL002041	-4.7	-4.43	7.69	9.52

## Concluding remarks

These results contribute to the understanding of the physiological response of *Ae*. *aegypti* larvae to an intoxication with an EEO, and allow to pose hypotheses for future physiological research in order to elucidate this response in a more detailed way. The results suggest that the detoxicant response to a natural plant compound is similar to that for most synthetic insecticides (i.e the involvement of CYPs, GSTs, HSPs, ABCs, UGTs or cuticular components), even though the expression of members of CCE family was not affected. Our results, in the context of previous literature, suggest that most gene families affected by different toxics are similar, even though particular members of each gene family are modulated in response to one or two toxics and not to others. This reinforces the hypothesis of a complex and versatile transcriptomic response in *Ae*. *aegypti* larvae after intoxication. Finally, our work provides important information regarding the implication of *Ae*. *aegypti* CSPs in the detoxification of a natural larvicide. Further experiments should include functional analysis of particular genes, evaluation of enzymatic activities and/or the study of changes in protein levels, in order to confirm or discard hypothesis suggested here by transcriptomic and docking data.

It is important to consider that the similarities in detoxificant responses to synthetic and natural insecticides could suggest that cross-resistance to both kinds of insecticides can take place in natural populations. The information provided here could be useful for further studies focused on the implementation of EOs to complement and/or replace synthetic insecticides in the control of mosquito populations.

The study and characterization of insect detoxification processes could be relevant for a rational design of pest control strategies. In this sense, the use of inhibitors of specific detoxification pathways could help to overcome resistance or low sensitivity of pest insects to insecticides. This could make Eucalyptus distilled compounds an interesting alternative into an integrated vector management of *Ae*. *aegypt*i.

## Supporting information

S1 Table Sequencing and mapping metrics. The data is expressed in millions paired end reads. C: control samples, T: treated samples
(XLSX)

S2 Table Effect of EEO treatment on the gene transcription. Those DEGs with an FDR<0.05 and a minimum 2-fold change threshold between control and exposed groups, are listed. One-hundred seventy seven overexpressed transcripts (red) and 62 underexpressed transcripts (blue) are included. The GO-terms of the *Ae*. *aegypti* predicted proteins were obtained from Vector Base using the BioMart tool. The longest isoform of the DEGs was chosen for the Blast searches vs. the Insecta database in NCBI.
(XLSX)

S3 Table KEGG enriched pathways (FDR <0.05) of differentially expressed genes. Fisher’s exact test and FDR correction based on Benjamini and Hochberg method were used. This analysis was performed using KOBAS 3.0, which evaluates the significance of enrichment of pathways and can be accessed via https://kobas.cbi.pku.edu.cn.
(XLSX)

S1 Fig Principal component analysis. Uncolored figures: control; filled figures: treated.
(TIF)

S2 Fig Volcano plot ofEEO treatment effect on gene transcription. The DEGs with an FDR<0.05 and a minimum 2-fold change threshold between control and exposed groups are shown in color, over-transcribed genes are shown in red and under-transcribed genes are in blue.
(TIF)

S3 Fig Effect of EEO treatment on the transcription of genes related to xenobiotic detoxification. Heatplots of families related to detoxification: A. Heat Shock Proteins 20; B. Heat Shock Proteins 70; C. Cytochromes P450; D. UDP-glycosyltransferases; E. Glutathione transferases; F. ABC transporters; G. Chemosensory proteins. Whenever a unique gene name was assigned in *Ae*. *aegypti* genome (www.vectorbase.org), this name is presented between brackets. Gene expression is represented as log2-counts per million reads (log-CPM) in which blue/red represent lowest/highest expression. Genes are identified by their vectorbase ID. Dendrogram was plotted with hierarchical clustering among samples and genes based on Euclidean distances and complete linkage method for clustering. C: control samples. T: treated samples. # = FDR<0.1; * = FDR<0.05; ** = FDR<0.01; *** = FDR<0.001.
(TIF)
