# Supplementary figures and images for: Transcriptomic analysis and molecular docking reveal genes involved in the response of Aedes aegypti larvae to an essential oil extracted from Eucalyptus
Source: PLoS Negl Trop Dis. 2021 Jul 16;15(7):e0009587. doi: 10.1371/journal.pntd.0009587 (PMC8318226; doi:10.1371/journal.pntd.0009587)

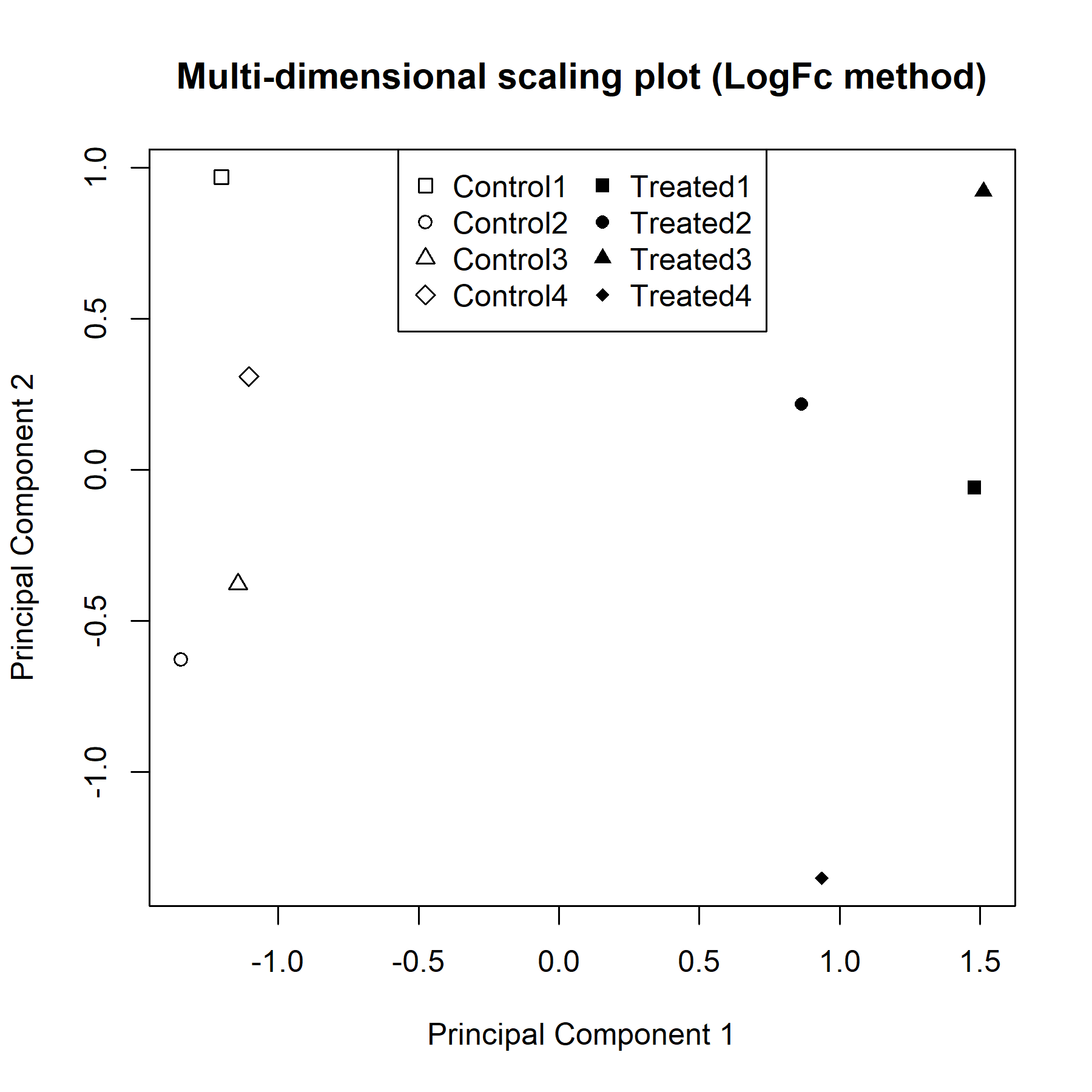

Supplement: S1 Fig — Uncolored figures: control; filled figures: treated. (TIF) [file pntd.0009587.s004.tif]

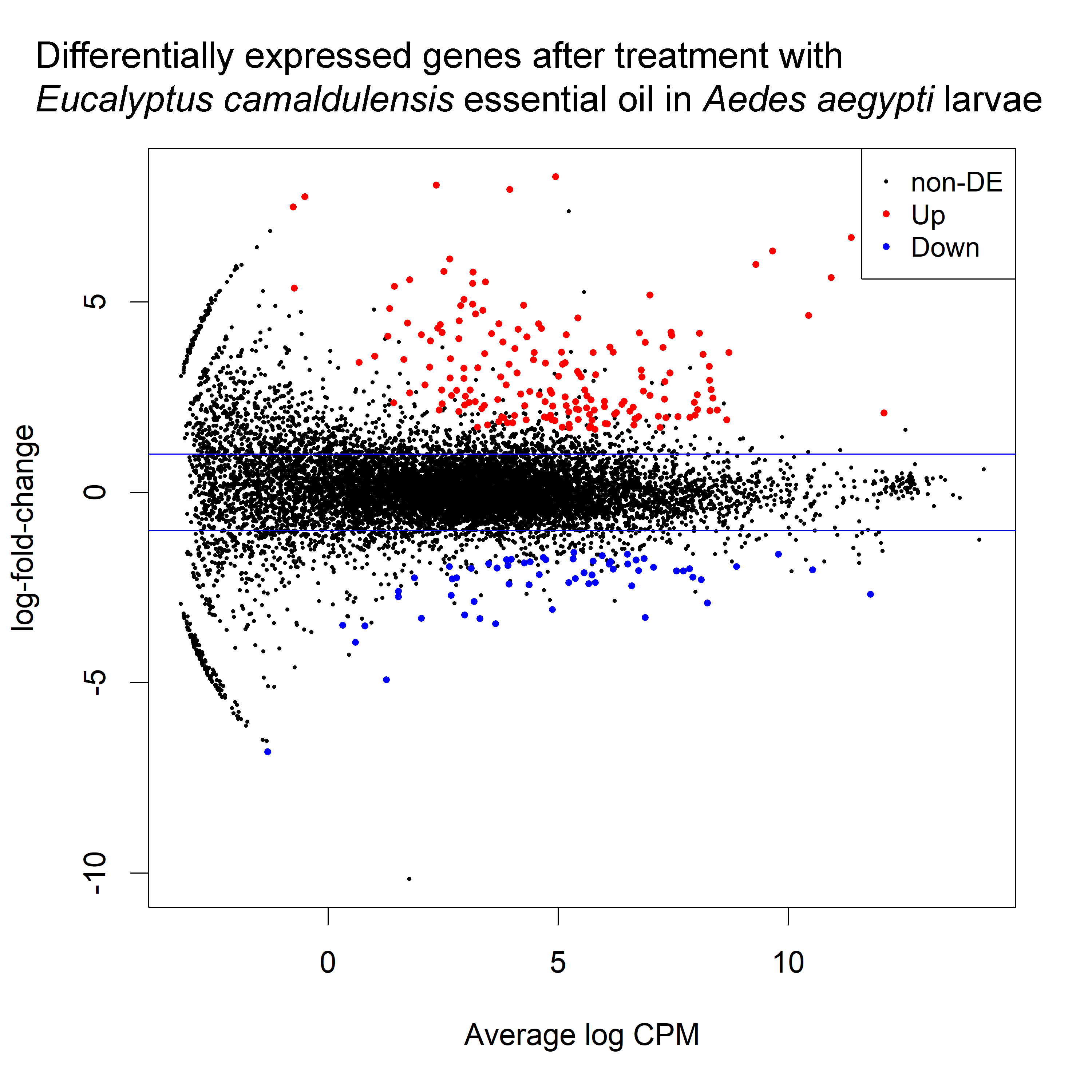

Supplement: S2 Fig — The DEGs with an FDR<0.05 and a minimum 2-fold change threshold between control and exposed groups are shown in color, over-transcribed genes are shown in red and under-transcribed genes are in blue. (TIF) [file pntd.0009587.s005.tif]

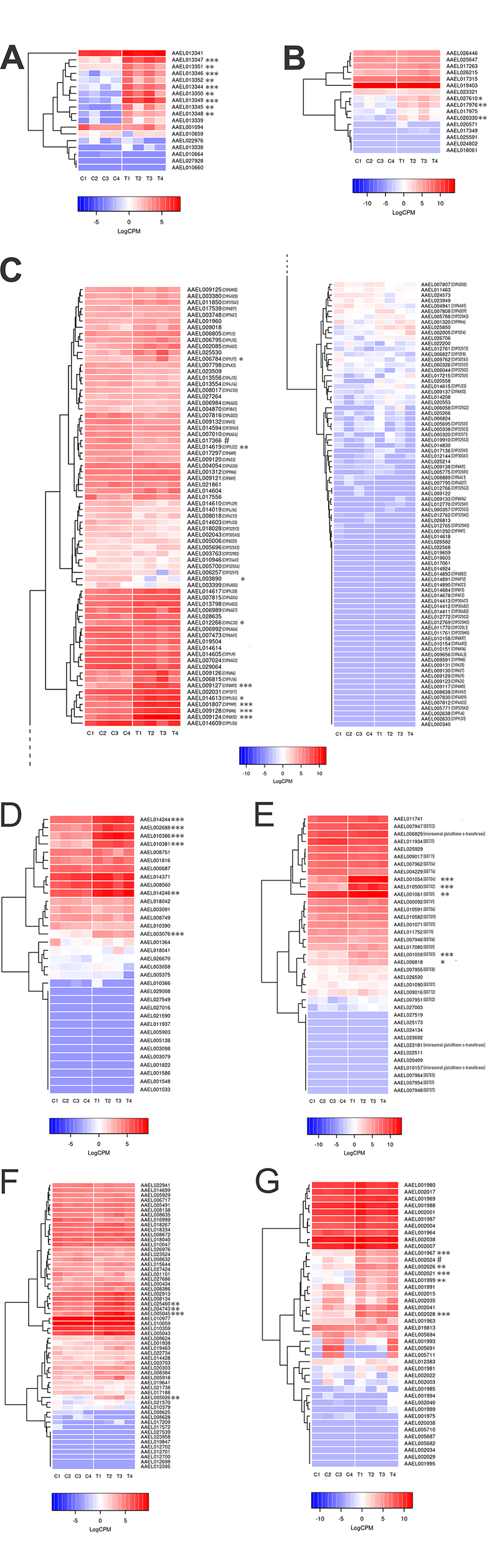

Supplement: S3 Fig — Heatplots of families related to detoxification: A. Heat Shock Proteins 20; B. Heat Shock Proteins 70; C. Cytochromes P450; D. UDP-glycosyltransferases; E. Glutathione transferases; F. ABC transporters; G. Chemosensory proteins. Whenever a unique gene name was assigned in Ae. aegypti genome (www.vectorbase.org), this name is presented between brackets. Gene expression is represented as log2-counts per million reads (log-CPM) in which blue/red represent lowest/highest expression. Genes are identified by their vectorbase ID. Dendrogram was plotted with hierarchical clustering among samples and genes based on Euclidean distances and complete linkage method for clustering. C: control samples. T: treated samples. # = FDR<0.1; * = FDR<0.05; ** = FDR<0.01; *** = FDR<0.001. (TIF) [file pntd.0009587.s006.tif]
